# Supplementary material for: Unstructured spare time as an international predictor of adolescent crime
Source: PLoS One. 2026 May 15;21(5):e0349291. doi: 10.1371/journal.pone.0349291 (PMC13178864; doi:10.1371/journal.pone.0349291)
Supplement: S1 File — (DOCX) [file pone.0349291.s001.docx]

**Supporting information for**

Unstructured spare time as an international predictor of adolescent crime

Appendices S1-S9

**Appendix S1. Sampling design and data collection**

The International Self-Report Delinquency Study (ISRD4) is a multinational survey designed to examine self-reported offending, victimization, and related social and psychological factors among adolescents. The fourth sweep of the study builds on the foundations established in previous rounds [1-4], maintaining its commitment to methodological rigor and cross-national comparability while adapting to contemporary challenges in school-based research. Launched in the 1990s, the ISRD study has progressed through three sweeps: ISRD1 (13 countries), ISRD2 (31 countries), and ISRD3 (36 countries), each expanding geographical scope, improving methodological harmonization, and revising key items, including those used in this study to measure structured and unstructured time use. Data collection in ISRD4 combined both probability school-based and non-probability internet-based samples to maximize reach and ensure inclusion of diverse adolescent populations. Our study only uses the probability school-based sample.

The target population for ISRD4 comprised adolescents aged 13 to 17. The primary sampling design involved a multistage clustered approach, focusing on urban populations. In each participating country, two large cities or metropolitan areas were selected, typically with populations over 500,000, though smaller countries could choose their most prominent cities. Within each city, a random sample of schools was drawn, ideally using probability proportional to size methods. From the sampled schools, entire classes were selected, and all students who were present on the day of the survey and who provided assent (alongside appropriate parental consent, where required) were invited to participate. The goal for each national team was to collect responses from at least 1,800 school-based students, evenly distributed across the two selected cities. Across the 21 countries included in our analysis, the final sample includes 58,425 adolescents drawn from 1,108 schools. Overall, the school access rate was 45.8%, although it varied considerably across countries, from a minimum of 6.7% in the UK to a maximum of 90.0% in Iceland. The student response rate was 70.5%, also with substantial variation across countries, ranging from 32.1% in Sweden to 98.8% in Argentina. Further details about the sampling design and data collection employed in each country can be found in Table S1.

The school-based survey instrument was administered using computer-assisted methods within the classroom setting, and the sample was constructed to ensure coverage across the target age range. In Argentina and Venezuela, the survey was conducted using paper and pencil due to limited digital infrastructure. In Slovenia and Bosnia and Herzegovina, a subset of students also completed paper-based surveys. The sampling aimed to include 225 students per age group (13-14, 14-15, 15-16, and 16-17) in each city, resulting in a total of 900 respondents per city. Age was the key inclusion criterion rather than grade, due to international variation in school systems. In some instances, pupils marginally younger or older than the target age range may have participated in the survey if they were enrolled in classes typically attended by students aged 13 to 17. National teams were permitted to use stratified sampling, for instance to oversample disadvantaged areas.

The ISRD4 survey instrument included a set of core questions that were mandatory across all countries, sweep-specific items unique to ISRD4, and optional national modules tailored to local priorities. The questionnaire was translated into national and minority languages using a structured and collaborative process that emphasized semantic and conceptual equivalence [4]. Pilot testing in each language ensured clarity and cultural appropriateness. Surveys were conducted in 21 languages, including national and minority languages, with English versions also available in most countries.

Ethical safeguards were integral to the study design. Participation was voluntary, and students were informed that they could skip any questions or withdraw from the survey at any time. Each national team was required to document the sampling process, school and student response rates, and any deviations from the protocol [4]. Data management was supported by a dedicated software tool (“Survey Manager”) that assisted with sampling logistics and monitoring. Data were collected between January 2021 and May 2024, with timelines varying across countries. Once data collection was complete, responses to the core and sweep-specific items were integrated into a central international dataset. Importantly, because samples were drawn from urban areas rather than nationally representative populations, cross-national comparisons should be interpreted as comparisons between cities rather than between entire countries.

**Table S1.** **Sampling design and data collection across participant countries.**

|  | Sample size | Number schools | Survey mode | School access rate | Student response rate | Survey dates | Survey languages |
| --- | --- | --- | --- | --- | --- | --- | --- |
| Argentina | 2,467 | 22 | Paper and pencil | 30.1 | 98.8 | Aug 2022 - Jun 2023 | Spanish (all) |
| Austria | 2,331 | 45 | Computer-assisted | 41.3 | Not reported | May 2022 - Dec 2022 | German (all) |
| Bosnia and Herz. | 1,858 | 44 | Computer-assisted (124 in paper and pencil) | 67.6 | 71.8 | Apr 2022 - Dec 2022 | Bosnian (1,766), Croatian (80), English (7), Serbian (5) |
| Brazil | 1,956 | 47 | Computer-assisted | 78.3 | 72.7 | Sep 2022 - Dec 2022 | Portuguese (1,946), English (10) |
| Colombia | 1,803 | 20 | Computer-assisted | Not reported | Not reported | May 2023 - Jun 2023 | Spanish (1,749), English (54) |
| Czech Republic | 2,212 | 62 | Computer-assisted | 51.7 | 80.5 | Feb 2023 - Jun 2023 | Czech (2,121), Ukrainian (52), Russian (27), English (12) |
| Denmark | 1,137 | 13 | Computer-assisted | 52.0 | Not reported | Sep 2022 - Dec 2022 | Danish (1,128), English (9) |
| Estonia | 6,404 | 61 | Computer-assisted | 67.0 | 82.5 | Apr 2023 - Jun 2023 | Estonian (4,735), Russian (1,635), Ukrainian (18), English (16) |
| Finland | 2,050 | 66 | Computer-assisted | 79.5 | 80.5 | Feb 2022 - Jun 2022 | Finnish (1,771), Swedish (142), English (137) |
| Iceland | 3,036 | 53 | Computer-assisted | 90.0 | 87.0 | Oct 2022 - Feb 2023 | Icelandic (2,899), English (137) |
| Lithuania | 1,914 | 33 | Computer-assisted | 39.0 | 74.9 | May 2022 - Jun 2022 | Lithuanian (1,898), English (16) |
| Mexico | 2,602 | 74 | Computer-assisted | 62.5 | 56.0 | May 2023 - Feb 2024 | Spanish (2,436), English (166) |
| Norway | 1,598 | 30 | Computer-assisted | 56.4 | 89.5 | Jun 2022 - Nov 2022 | Norwegian (1,570), English (28) |
| Poland | 2,209 | 51 | Computer-assisted | Not reported | Not reported | Dec 2023 - Jan 2024 | Polish (all) |
| Slovenia | 2,649 | 39 | Computer-assisted (797 in paper and pencil) | 79.5 | 71.2 | Oct 2022 - Mar 2023 | Slovene (2,563), English (86) |
| Spain | 1,268 | 14 | Computer-assisted | 9.5 | 86.5 | May 2022 - Mar 2023 | Spanish (1,255), English (13) |
| Sweden | 1,414 | 39 | Computer-assisted | 48.4 | 32.1 | Mar 2023 - May 2023 | Swedish (1,369), English (45) |
| Switzerland | 11,880 | 323 | Computer-assisted | 66.2 | 51.1 | Jan 2021 - June 2022 | French (5,672), German (5,621), Italian (587) |
| UK | 3,866 | 46 | Computer-assisted | 6.7 | 62.6 | Nov 2022 - Jun 2023 | English (all) |
| USA | 2,061 | 3 | Computer-assisted | Not reported | Not reported | May 2023 - May 2024 | English (all) |
| Venezuela | 1,710 | 23 | Paper and pencil | 88.9 | 97.7 | Dec 2022 - Dec 2022 | Spanish (all) |

Appendix S2. Conceptualizing unstructured spare time and its relationship to adolescent offending

The concept of unstructured spare time refers to time spent on activities for amusement, relaxation, or entertainment without predetermined agendas or goals. This conceptualization extends beyond Osgood et al.’s notion of unstructured socializing [5], as it includes all leisure activities that are unscheduled or unplanned, regardless of whether they involve peers. This expanded approach is theoretically and empirically justified for several reasons. First, although much prior research has emphasized unstructured peer-based socializing [e.g., 6-9], both solitary and social forms of unstructured time may expose young people to criminogenic situations [10]. Second, adolescents now spend substantial amounts of time alone in digital environments, creating extended periods of unstructured activity without direct peer interaction, during which opportunities for risk-taking can still arise in the absence of structured routines. Third, engagement in unstructured activities—whether alone or with others—necessarily displaces participation in structured, supervised, and socially integrated activities that are linked to lower offending and stronger prosocial development [11-13]. Finally, restricting the concept to peer-based socializing risks placing excessive emphasis on adolescence while overlooking criminogenic processes that may operate earlier in childhood (e.g., unsupervised time spent alone) or later in adulthood (e.g., unstructured time associated with substance use or gambling). Taken together, unstructured spare time offers a more comprehensive lens for examining how everyday routines shape exposure to risk across the life course.

Unstructured spare time can facilitate offending not simply by increasing the amount of time available for deviant behavior, but also by shaping situational contexts in ways that reduce supervision and increase criminal opportunity [5, 9, 10]. In addition, research shows that unstructured time spent with peers can foster greater tolerance for offending and substance use, reinforcing norms and motivations that support delinquent behavior [14]. These processes indicate that unstructured spare time creates situational conditions that are conducive to crime, even in the absence of immediate criminal intent. The present study therefore examines whether this broader time-use exposure shows a consistent association with adolescent offending across diverse national contexts, providing a foundation for theory refinement on how individual dispositions, social environments and opportunity structures interact to shape youth crime.

Appendix S3. Confirmatory Factor Analysis of self-control, parental control, exposure to crime, and morality

Figure S1 presents the results of four confirmatory factor analyses (CFAs) conducted to validate the latent constructs used as controls in the study. All models demonstrate good fit, with high Comparative Fit Index (CFI) and Tucker-Lewis Index (TLI), and low Root Mean Square Error of Approximation (RMSEA), supporting the reliability of the measurement models.

The latent variable ‘self-control’ is measured by four items reflecting impulsivity, hedonistic tendencies, short-term orientation, and risk-taking [15-17], as these are the most frequently considered measures of self-control in crime research [18]. The scale is an abbreviated version of Grasmick et al. [17]. Standardized factor loadings range from 0.48 to 0.71, with the strongest loading for “I do whatever brings me pleasure here and now, even at the cost of some future goal.” The model fit indices (CFI = 0.997, TLI = 0.991, RMSEA = 0.029) indicate excellent model fit.

The latent construct ‘parental control’ is measured by four indicators capturing parental support, monitoring, and supervision. These include awareness of the adolescent's whereabouts and online activities, as well as perceived emotional support. Items are derived from the Parental Knowledge Scale [19]. Factor loadings range from 0.34 to 0.74, with the highest loading observed for “an adult at home knows what I am doing when I go out.” Model fit is acceptable (CFI = 0.978, TLI = 0.935, RMSEA = 0.079).

The latent factor ‘exposure to crime’ is reflected in perceptions of crime in the neighborhood and school environment. Loadings range from 0.32 to 0.75, indicating moderate to strong associations between the indicators and the latent construct. The five items include being aware of crime in the neighborhood, as well as perceptions of theft, fighting, vandalism, and drug use at school. The model demonstrates excellent fit (CFI = 0.995, TLI = 0.990, RMSEA = 0.033).

The construct ‘morality’ is assessed through six items measuring the perceived wrongfulness of various deviant and criminal behaviors, including hate speech, property damage, physical violence, and cyber offences [9, 20, 21]. The scale is an abbreviated version of the tests of Situational Action Theory [9]. Loadings range from 0.62 to 0.75, indicating strong internal consistency. The fit indices (CFI = 0.985, TLI = 0.975, RMSEA = 0.057) support a robust model.

For each latent factor, score estimates were computed using the Full Information Maximum Likelihood (FIML) estimator, maximizing the use of available data for each respondent and inferring missing values based on observed data. All scores were rescaled to a 0-10 positive range to facilitate interpretation: $\frac{F_{i}-min(F)}{\max\left( F \right)-min(F)}\times10$, where $F_{i}$ is the latent score for unit $i$.

| 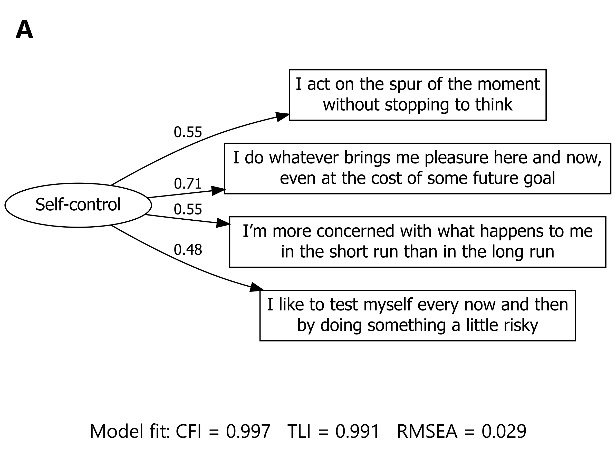 | 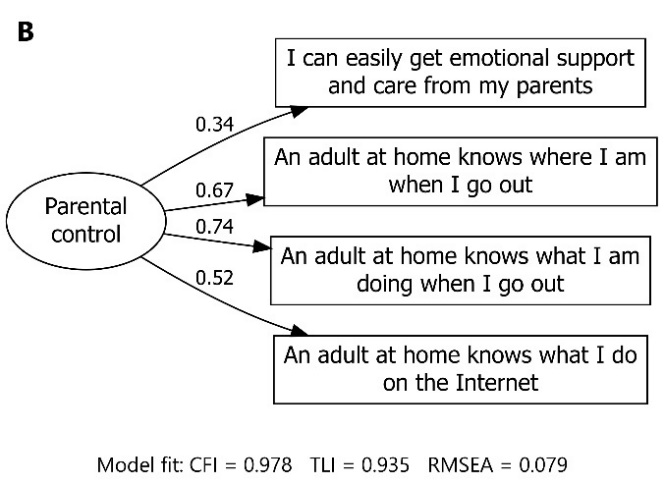 |
| --- | --- |
| 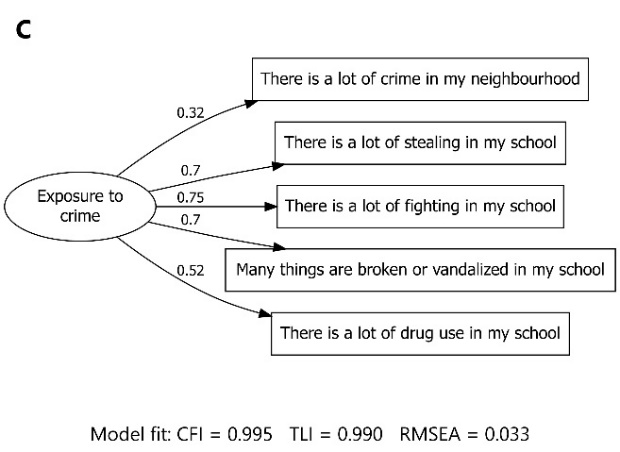 | 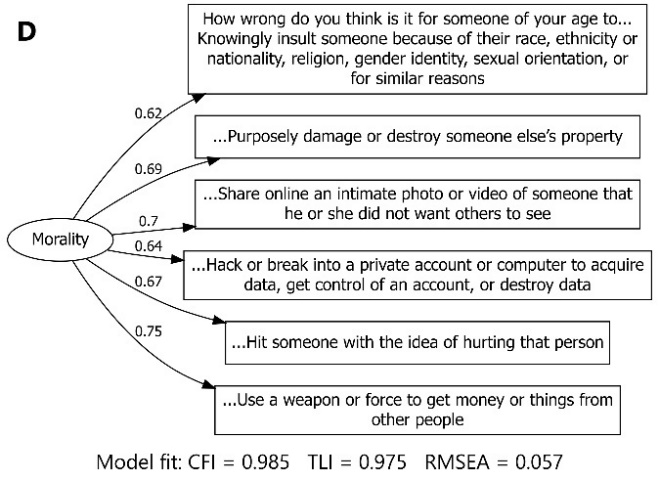 |

Fig S1. Confirmatory Factor Analysis of (A) self-control, (B) parental control, (C) exposure to crime and (D) morality.

Appendix S4. Descriptive statistics of key dependent, independent and control variables

Table S2. Descriptive statistics of self-reported offending variables, time use variables, key criminological variables, and control variables.

|  |  | | Min | 1^st^ Q | Mean (SD) | Median | 3^rd^ Q | Max |
| --- | --- | --- | --- | --- | --- | --- | --- | --- |
| Self-reported offending variables | Drug crime | Prevalence | 0 | 0 | 0.0 (0.2) | 0 | 0 | 1 |
|  |  | Incidence | 0 | 0 | 0.1 (1.2) | 0 | 0 | 60 |
|  | Violence | Prevalence | 0 | 0 | 0.1 (0.2) | 0 | 0 | 1 |
|  |  | Incidence | 0 | 0 | 0.2 (1.1) | 0 | 0 | 42 |
|  | Damage | Prevalence | 0 | 0 | 0.1 (0.3) | 0 | 0 | 1 |
|  |  | Incidence | 0 | 0 | 0.3 (1.5) | 0 | 0 | 45 |
|  | Property crime | Prevalence | 0 | 0 | 0.1 (0.3) | 0 | 0 | 1 |
|  |  | Incidence | 0 | 0 | 0.4 (2.1) | 0 | 0 | 65 |
|  | Cyber crime | Prevalence | 0 | 0 | 0.1 (0.3) | 0 | 0 | 1 |
|  |  | Incidence | 0 | 0 | 0.4 (2.5) | 0 | 0 | 84 |
|  | All crime | Prevalence | 0 | 0 | 0.2 (0.4) | 0 | 0 | 1 |
|  |  | Incidence | 0 | 0 | 1.3 (5.1) | 0 | 0 | 210 |
| Time use variables | Unstructured out-of-home spare time | | 0 | 0.2 | 0.7 (0.7) | 0.5 | 1.0 | 5.5 |
|  | Unstructured spare time at home | | 0 | 2.0 | 6.0 (3.8) | 7.2 | 8.6 | 18.2 |
|  | Structured spare time at home | | 0 | 2.2 | 3.6 (2.2) | 3.3 | 4.7 | 11.7 |
| Criminological variables | Self-control^1^ | | 0 | 3.5 | 5.0 (2.2) | 5.0 | 6.5 | 10.0 |
|  | Parental control^1^ | | 0 | 6.8 | 7.8 (1.9) | 8.3 | 9.3 | 10.0 |
|  | Morality^1^ | | 0 | 8.5 | 8.9 (1.5) | 9.5 | 9.9 | 10.0 |
|  | Delinquent peers (binary) | | 0 | 0 | 0.3 (0.5) | 0 | 1 | 1 |
|  | Exposure to crime^1^ | | 0 | 2.1 | 3.7 (2.2) | 3.6 | 5.2 | 10.0 |
| Control variables | Male (binary) | | 0 | 0 | 0.5 (0.5) | 0 | 1 | 1 |
|  | Age | | 11 | 14 | 15.1 (1.3) | 15 | 16 | 21 |
|  | Born in country (binary) | | 0 | 1 | 0.9 (0.3) | 1 | 1 | 1 |
|  | Family deprivation (ordinal) | | 1 | 2 | 3.2 (1.2) | 3 | 4 | 7 |
|  | Openness (ordinal) | | 1 | 3 | 3.0 (0.9) | 3 | 4 | 4 |

^1^Factor score estimates rescaled to 0-10 positive range to facilitate interpretation.

Appendix S5. Country-level means of self-reported offending and structured and unstructured spare time

Table S3. Country-level means of self-reported offending and structured and unstructured spare time (standard deviations in parentheses).

|  | Unstructured out-of-home spare time | Unstructured spare time at home | Structured spare time at home | Non-spare time | Offending incidence | Offending prevalence |
| --- | --- | --- | --- | --- | --- | --- |
| Argentina | 0.80 (0.72) | 7.62 (3.30) | 4.43 (2.72) | 11.16 (2.89) | 1.62 (4.23) | 0.30 (0.46) |
| Austria | 0.60 (0.64) | 6.37 (3.84) | 3.26 (1.90) | 13.78 (3.35) | 1.52 (4.85) | 0.22 (0.41) |
| Bosnia and Herz. | 0.91 (0.83) | 6.41 (4.01) | 3.30 (2.00) | 13.39 (3.38) | 0.94 (3.85) | 0.18 (0.38) |
| Brazil | 0.77 (0.80) | 4.68 (3.58) | 4.49 (2.55) | 14.06 (3.48) | 0.85 (2.76) | 0.18 (0.38) |
| Colombia | 0.53 (0.57) | 6.58 (3.66) | 5.52 (2.86) | 11.38 (3.23) | 0.69 (2.32) | 0.16 (0.37) |
| Czech Republic | 0.80 (0.69) | 5.75 (3.94) | 2.57 (1.94) | 14.88 (3.48) | 1.75 (5.64) | 0.25 (0.43) |
| Denmark | 0.49 (0.56) | 6.54 (3.93) | 3.06 (1.58) | 13.91 (3.27) | 0.85 (3.33) | 0.16 (0.37) |
| Estonia | 0.79 (0.68) | 6.48 (3.96) | 3.09 (1.91) | 13.64 (3.48) | 1.51 (7.22) | 0.18 (0.38) |
| Finland | 0.50 (0.53) | 6.13 (3.60) | 3.14 (1.69) | 14.23 (3.09) | 0.98 (3.80) | 0.17 (0.37) |
| Iceland | 0.60 (0.62) | 5.44 (3.65) | 3.63 (1.85) | 14.33 (3.20) | 1.27 (5.17) | 0.18 (0.39) |
| Lithuania | 1.07 (0.77) | 5.64 (3.87) | 3.28 (1.85) | 14.02 (3.38) | 0.73 (2.76) | 0.17 (0.37) |
| Mexico | 0.69 (0.62) | 6.89 (3.39) | 4.85 (2.61) | 11.57 (3.23) | 1.04 (3.60) | 0.19 (0.39) |
| Norway | 0.58 (0.56) | 5.97 (3.52) | 3.65 (2.17) | 13.80 (3.08) | 0.92 (3.14) | 0.17 (0.38) |
| Poland | 0.73 (0.69) | 5.89 (4.27) | 3.57 (2.23) | 13.81 (3.73) | 1.22 (5.21) | 0.16 (0.37) |
| Slovenia | 0.77 (0.71) | 5.14 (3.96) | 3.51 (1.80) | 14.57 (3.54) | 1.41 (5.40) | 0.20 (0.40) |
| Spain | 1.00 (0.68) | 6.22 (3.48) | 4.35 (2.44) | 12.43 (3.05) | 1.40 (4.07) | 0.29 (0.45) |
| Sweden | 0.61 (0.60) | 6.14 (3.81) | 3.08 (1.64) | 14.17 (3.36) | 0.58 (2.41) | 0.13 (0.34) |
| Switzerland | 0.63 (0.66) | 5.58 (3.83) | 3.46 (1.65) | 14.32 (3.25) | 1.99 (7.04) | 0.28 (0.45) |
| UK | 0.60 (0.58) | 6.75 (3.93) | 3.10 (2.07) | 13.56 (3.36) | 0.83 (3.60) | 0.14 (0.34) |
| USA | 0.45 (0.60) | 7.04 (4.04) | 3.26 (2.35) | 13.24 (3.71) | 0.65 (2.52) | 0.13 (0.34) |
| Venezuela | 0.63 (0.72) | 5.25 (3.75) | 5.91 (2.75) | 12.21 (3.40) | 0.52 (1.82) | 0.15 (0.36) |

Appendix S6. Analysis of unstructured spare time among respondents with zero, low-frequency, and high-frequency self-reported offending, as defined by the 95th percentile of self-reported offences across the pooled national samples

As a robustness check for the results presented in Fig 1, we replicated the analysis using an alternative classification of ‘low frequency’ and ‘high frequency’ offenders, based on the 95th percentile of self-reported offences across the *pooled* national samples (i.e., 7 offences). The results remained remarkably consistent, as displayed in Fig S2. High-frequency offenders reported more unstructured spare time than non-offenders in every country, with the difference statistically significant at the p < 0.05 level in all countries except Sweden and the USA. Similarly, low frequency offenders reported higher average levels of unstructured spare time than non-offenders across all countries, although the difference was not statistically significant in Sweden. High-frequency offenders also reported more unstructured spare time than low frequency offenders, though this difference did not reach statistical significance in eight countries (Argentina, Brazil, Denmark, Lithuania, Norway, Sweden, USA, and Venezuela). On average, non-offenders reported spending 6.4 hours per day in unstructured spare time activities, compared to 7.5 hours for low frequency offenders and 8.8 hours for high frequency offenders.


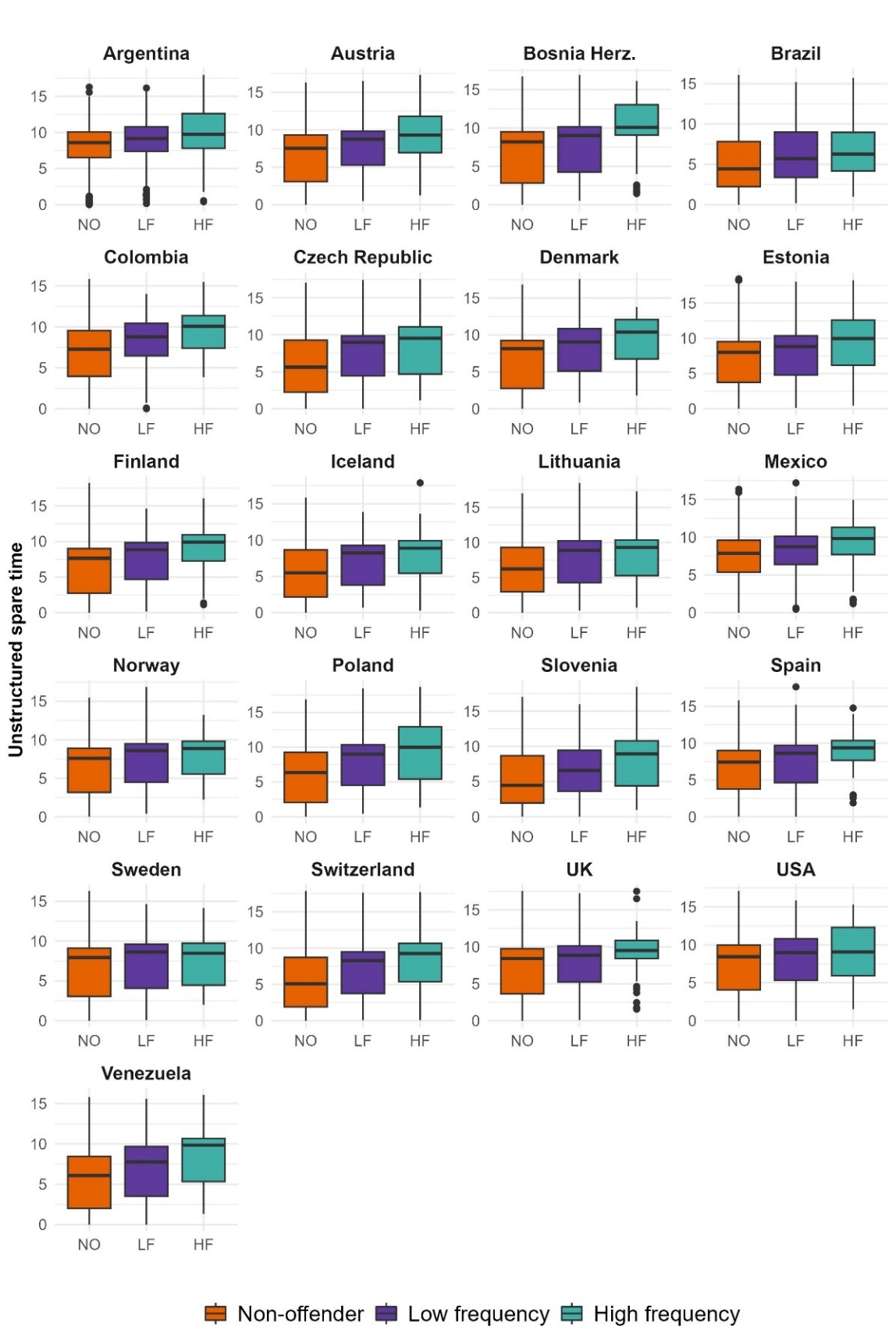


Fig S2. Boxplots of unstructured spare time (out-of-home and at home) among respondents with zero, low-frequency, and high-frequency self-reported offending, as defined by the 95th percentile of self-reported offences across the pooled national samples.

Appendix S7. Negative Binomial regression for self-reported crime counts

As a robustness check, we re-estimated the offending incidence model using a Negative Binomial specification to assess sensitivity to the choice of count model. As shown in Table S4, the Negative Binomial estimates are substantively equivalent to those obtained from the quasi-Poisson model, with all key predictors retaining similar magnitude of estimates, directions, and levels of statistical significance.

**Table S4. Comparison of quasi-Poisson (main manuscript) and Negative Binomial models of self-reported offending.**

|  | Quasi-Poisson (original) | | | Negative Binomial | | |
| --- | --- | --- | --- | --- | --- | --- |
|  | IRR | 95% CI | p-value | IRR | 95% CI | p-value |
| (Intercept) | **0.40** | 0.34-0.47 | <0.001 | **0.37** | 0.31-0.44 | <0.001 |
| Unstructured out-of-home spare time | **1.70** | 1.62-1.77 | <0.001 | **1.78** | 1.68-1.89 | <0.001 |
| Unstructured spare time at home | **1.29** | 1.21-1.37 | <0.001 | **1.47** | 1.38-1.57 | <0.001 |
| Structured spare time at home | **0.78** | 0.72-0.84 | <0.001 | **0.82** | 0.77-0.88 | <0.001 |
| Self-control | **0.61** | 0.58-0.64 | <0.001 | **0.58** | 0.54-0.61 | <0.001 |
| Parental control | **0.68** | 0.65-0.71 | <0.001 | **0.55** | 0.52-0.58 | <0.001 |
| Morality | **0.62** | 0.60-0.65 | <0.001 | **0.47** | 0.44-0.50 | <0.001 |
| Delinquent peers | **4.39** | 4.12-4.69 | <0.001 | **4.51** | 4.24-4.79 | <0.001 |
| Exposure to crime | **1.34** | 1.27-1.41 | <0.001 | **1.45** | 1.36-1.55 | <0.001 |
| Gender (male) | **1.63** | 1.54-1.72 | <0.001 | **1.87** | 1.76-1.98 | <0.001 |
| Age | **0.92** | 0.86-0.97 | 0.010 | **0.90** | 0.85-0.96 | 0.022 |
| Born in country | 0.94 | 0.87-1.02 | 0.230 | 1.01 | 0.92-1.12 | 0.827 |
| Family deprivation | 1.02 | 0.97-1.07 | 0.626 | 0.97 | 0.92-1.03 | 0.503 |
| Openness (sincerity) | **1.57** | 1.48-1.66 | <0.001 | **1.63** | 1.54-1.73 | <0.001 |
| Observations | 44,427 | | | 44,427 | | |
| Pseudo R^2^ Nagelkerke | 0.90 | | | 0.43 | | |

Standardized coefficients. Country fixed effects are included but not displayed. Robust p-values are based on standard errors clustered at the school level. Pseudo R^2^ values are provided for descriptive purposes but should not be interpreted as directly comparable across model families.

Appendix S8. Regression analysis for specific crime types

Fig S3 summarizes the estimated associations of three types of spare time (unstructured spare time at home (purple), unstructured out-of-home spare time (orange), and structured spare time at home (light blue)) with five types of self-reported offending: drug offences, violence, damage, property crime, and cybercrime. These associations are shown using odds ratios and incidence rate ratios and 95% confidence intervals from two types of models: binary logistic regression (for offending prevalence) and quasi-Poisson regression (for offending incidence).

Across all crime types and both model specifications, unstructured out-of-home spare time emerges as a consistently strong and statistically significant correlate of adolescent offending. Its estimated associations are particularly pronounced for drug offences, violence, damage and property crime, with odds ratios above 1.5. The association between unstructured out-of-home spare time and self-reported cybercrime incidence is statistically significant, though the estimated association is much smaller. This may reflect that cyber offending is less dependent on public or unsupervised physical settings and is instead more closely tied to parental control over unstructured internet use [22, 23]. Unstructured spare time at home is also significantly associated with most types of offending but not drug crimes, though its estimated associations are generally smaller. In contrast, structured spare time at home generally shows negative associations with self-reported offending; especially in relation to damage, cybercrime, and property crime, but not violence. Regression estimates support the conclusion that time use is significantly associated with adolescents’ offending patterns, with unstructured out-of-home activities being positively associated with offending and structured time at home showing a negative association [13, 24, 25].


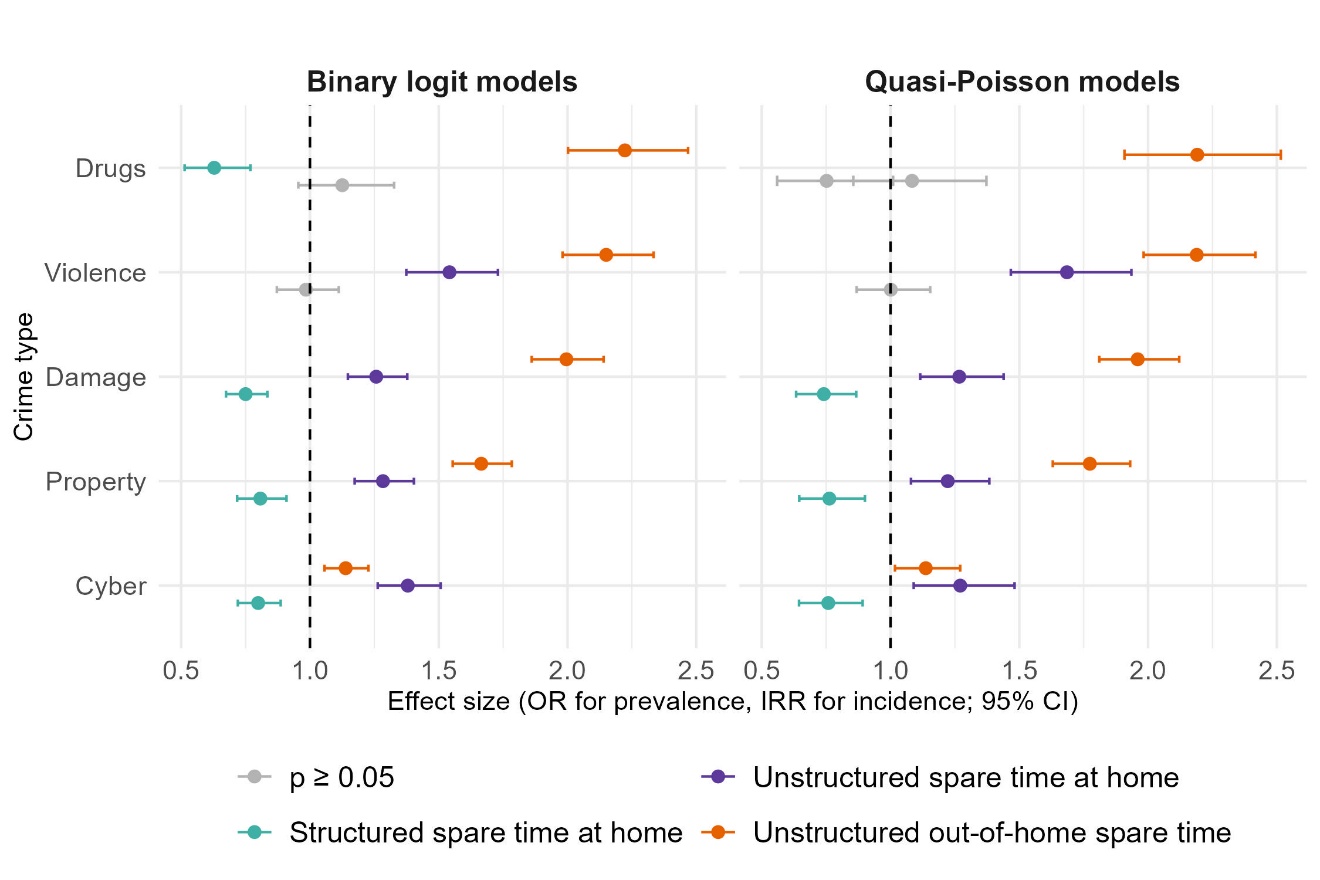


Fig S3. Regression analysis for specific crime types. Standardized coefficients. Country fixed effects are included but not displayed. Estimates adjust for all key criminological and control variables. Robust p-values and confidence intervals are based on standard errors clustered at the school level.

Appendix S9. Robustness check on main results after excluding truancy and navigating the darknet from unstructured spare time measures

To ensure that the observed associations are not driven by the inclusion of spare time activities that may themselves be considered deviant rather than merely unstructured, we re-estimated the main analyses after excluding truancy and navigating the darknet from the unstructured spare time measures. As shown in Table S5 and Fig S4, the results remain substantively equivalent to the main analyses, with all key associations retaining similar directions, magnitudes, and levels of statistical significance. Minor differences emerge in a small number of country-specific models; for example, the association between unstructured out-of-home spare time and self-reported offending incidence is no longer statistically significant in Spain, whereas this association becomes significant in Venezuela. Overall, the main results are robust to the exclusion of potentially deviant time-use items.

**Table S5. Binary logit model and quasi-Poisson model of self-reported offending (excluding truancy and navigating the darknet from unstructured spare time measures).**

|  | Model 1  Binary logit model | | | Model 2  Quasi-Poisson model | | |
| --- | --- | --- | --- | --- | --- | --- |
|  | OR | 95% CI | p-value | IRR | 95% CI | p-value |
| (Intercept) | **0.16** | 0.14-0.19 | <0.001 | **0.43** | 0.37-0.50 | <0.001 |
| Unstructured out-of-home spare time | **1.58** | 1.50-1.67 | <0.001 | **1.61** | 1.54-1.68 | <0.001 |
| Unstructured spare time at home | **1.28** | 1.20-1.36 | <0.001 | **1.25** | 1.17-1.33 | <0.001 |
| Structured spare time at home | **0.78** | 0.73-0.83 | <0.001 | **0.75** | 0.70-0.81 | <0.001 |
| Self-control | **0.56** | 0.53-0.60 | <0.001 | **0.62** | 0.58-0.65 | <0.001 |
| Parental control | **0.59** | 0.55-0.62 | <0.001 | **0.66** | 0.63-0.69 | <0.001 |
| Morality | **0.65** | 0.61-0.68 | <0.001 | **0.59** | 0.57-0.62 | <0.001 |
| Delinquent peers | **5.39** | 5.11-5.68 | <0.001 | **4.67** | 4.38-4.97 | <0.001 |
| Exposure to crime | **1.27** | 1.19-1.34 | <0.001 | **1.35** | 1.28-1.43 | <0.001 |
| Gender (male) | **1.40** | 1.32-1.47 | <0.001 | **1.65** | 1.56-1.74 | <0.001 |
| Age | **0.82** | 0.78-0.87 | <0.001 | **0.93** | 0.88-0.98 | 0.028 |
| Born in country | 1.02 | 0.94-1.12 | 0.607 | 0.94 | 0.87-1.02 | 0.240 |
| Family deprivation | **1.07** | 1.02-1.13 | 0.017 | 1.04 | 0.99-1.09 | 0.281 |
| Openness (sincerity) | **1.78** | 1.68-1.88 | <0.001 | **1.56** | 1.48-1.64 | <0.001 |
| Observations | 44,427 | | | 44,427 | | |
| χ^2^ test compared to null model | 10,834 (p<0.001) | | | 110,636 (p<0.001) | | |
| Pseudo R^2^ SSE | 0.33 | | | 0.34 | | |
| Pseudo R^2^ Nagelkerke | 0.44 | | | 0.90 | | |

Standardized coefficients. Country fixed effects are included but not displayed. Robust p-values are based on standard errors clustered at the school level. Pseudo R^2^ values are provided for descriptive purposes but should not be interpreted as directly comparable across model families.


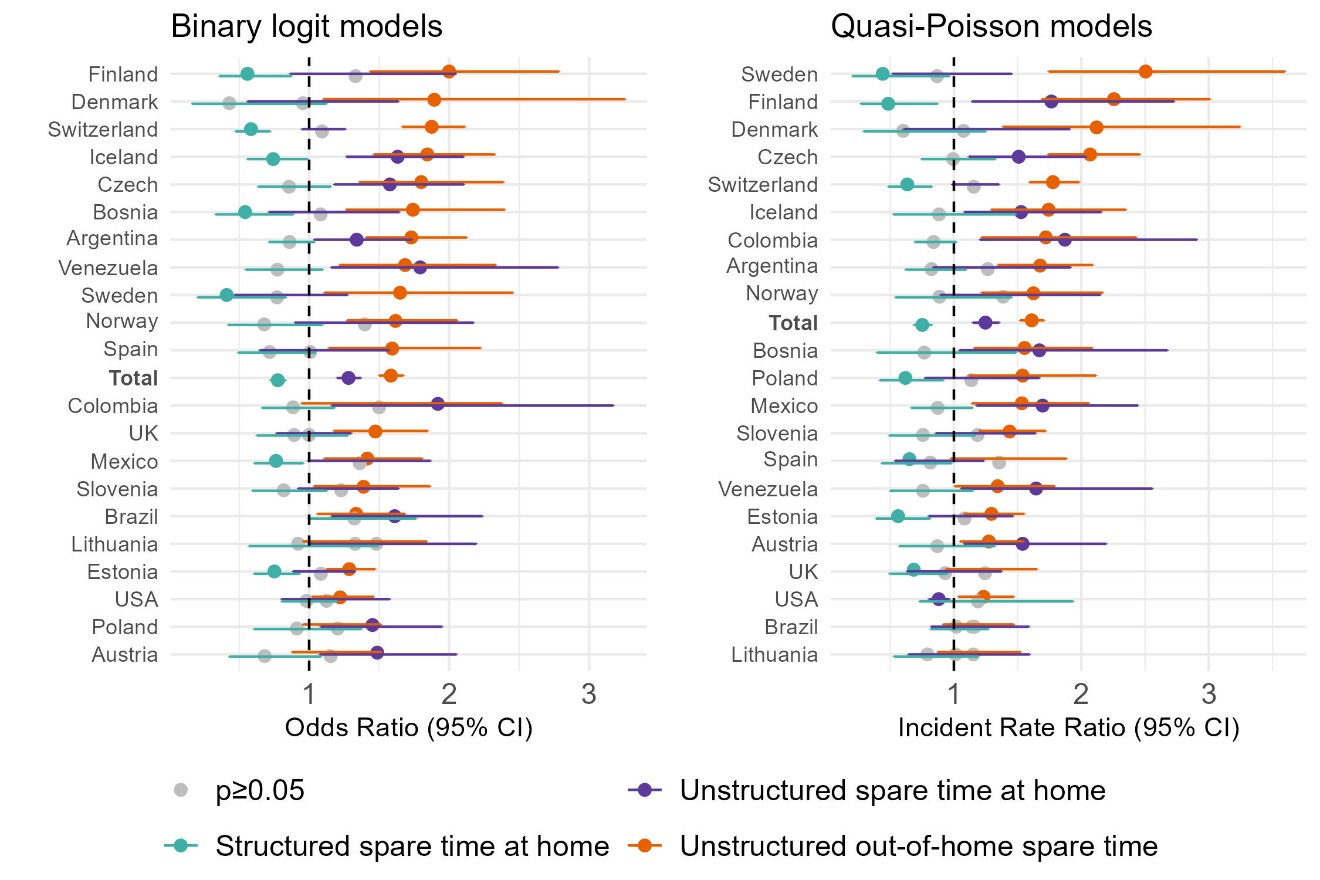


**Fig S4.** Logistic and quasi-Poisson regression models of spare time (excluding truancy and navigating the darknet) and self-reported offending in each national sample. Standardized coefficients. Estimates adjust for all key criminological and control variables. Robust p-values and confidence intervals are based on standard errors clustered at the school level.

**SI References**

1. Junger-Tas J. The significance of the International Self-report Delinquency Study (ISRD). Eur J Crim Policy Res. 2010 Jun;16:71-87.
2. Junger-Tas J, Marshall IH, Enzmann D, Killias M, Steketee M, Gruszczynska B. Juvenile delinquency in Europe and beyond: Results of the second International Self-Report Delinquency Study. Dordrecht: Springer; 2010.
3. Marshall IH, Neissl K, Markina A. A global view on youth crime and victimization: Results from the International Self-Report Delinquency Study (ISRD3). J Contemp Crim Just. 2019 Nov;35(4):380-5.
4. Marshall IH, Birkbeck C, Enzmann D, Kivivuori J, Markina A, Stekete M. International Self-Report Delinquency (ISRD4) study protocol: Background, methodology and mandatory items for the 2021/2022 survey. Boston: Northeastern University; 2022 [cited 2024 Dec 21]. Available from: <https://nbn-resolving.org/urn:nbn:de:0168-ssoar-78879-1>
5. Osgood DW, Wilson JK, O’Malley PM, Bachman JG, Johnston LD. Routine activities and individual deviant behavior. Am Sociol Rev. 1996 Aug;61(4):635-55.
6. Deitzer JR, McGloin JM, Hoeben E. Specifying the temporal bounds of the situational peer effect. J Res Crime Delinq. 2025 Aug;62(5):703-43.
7. Hoeben EM, Meldrum RC, Walker D, Young JTN. The role of peer delinquency and unstructured socializing in explaining delinquency and substance use: A state-of-the-art review. J Crim Justice. 2016 Dec;47:108-22.
8. Hoeben EM, Weerman FM. Why is involvement in unstructured socializing related to adolescent delinquency? Criminology. 2016 May;54(2):242-81.
9. Wikström PO, Oberwittler D, Treiber K, Hardie B. Breaking rules: The social and situational dynamics of young people's urban crime. Oxford: Oxford University Press; 2012.
10. Bernasco W, Ruiter S, Bruinsma GJ, Pauwels LJ, Weeman FM. Situational causes of offending: A fixed‐effects analysis of space–time budget data. Criminology. 2013 Nov;51(4):895-926.
11. Badura P, Madarasova Geckova A, Sigmundova D, Sigmund E, van Dijk JP, Reijneveld SA. Can organized leisure-time activities buffer the negative outcomes of unstructured activities for adolescents’ health? Int J Public Health. 2018 Jun 2;63(6):743-51.
12. Bone JK, Bu F, Fluharty ME, Paul E, Sonke JK, Fancourt D. Arts and cultural engagement, reportedly antisocial or criminalized behaviors, and potential mediators in two longitudinal cohorts of adolescents. J Youth Adolescence. 2022 Aug;51(8):1463-82.
13. Mahoney JL, Larson RW, Eccles JS, editors. Organized activities as contexts of development: Extracurricular activities, after-school and community programs. New York: Lawrence Erlbaum; 2005.
14. Hoeben EM, Weerman FM. Why Is involvement in unstructured socializing related to adolescent delinquency? Criminology. 2016 May;54(2):242-81.
15. Gottfredson MR, Hirschi T. A general theory of crime. Stanford: Stanford University Press; 1990.
16. Arneklev B, Grasmick H, Bursik R. Evaluating the dimensionality and invariance of “low self-control”. J Quant Criminol. 1999 Sep;15:307-31.
17. Grasmick H, Tittle C, Bursik R, Arneklev B. Testing the core empirical implications of Gottfredson and Hirschi’s General Theory of Crime. J Res Crime Delinq. 1993 Feb; 30(1):5-29.
18. Forrest W, Hay C, Widdowson A, Rocque M. Development of impulsivity and risk-seeking: Implications for the dimensionality and stability of self-control. Criminology. 2019 Aug;57(3):512-43.
19. Eaton NR, Krueger RF, Johnson WR, McGue M, Iacono WG. Parental monitoring, personality, and delinquency: Further support for a reconceptualization of monitoring. J Res Pers. 2009;43(1):49-59.
20. Wikström PO, Butterworth D. Adolescent crime: Individual differences and lifestyles. Cullompton: Willan Publishing; 2006.
21. Menard S, Elliott D. Delinquent bonding, moral beliefs, and illegal behavior: A three-wave panel model. Justice Q. 1994;11(2):173-88.
22. Nagata JM, Trompeter N, Singh G, Ganson KT, Testa A, Jackson DB, et al. Social epidemiology of early adolescent cyberbullying in the United States. Acad Pediatr. 2022 Noov;22(8):1287-93.
23. Wang W. Exploring the relationship among free-time management, leisure boredom, and internet addiction in undergraduates in Taiwan. Psychol Rep. 2019 Oct;122(5):1651-65.
24. Mahoney JL, Stattin H. Leisure activities and adolescent antisocial behavior: The role of structure and social context. J Adolescence. 2000 Apr;23(2):113-27.
25. Buil-Gil D. The structure of unstructured time and crime: A spare time model. Brit J Criminol. 2026 Jan;66(1):1-22.
