# Supplementary material for: Unstructured spare time as an international predictor of adolescent crime
Source: PLoS One. 2026 May 15;21(5):e0349291. doi: 10.1371/journal.pone.0349291 (PMC13178864; doi:10.1371/journal.pone.0349291)
Supplement: Attachment — Submitted filename: Response to Reviewers.docx [file pone.0349291.s002.docx]

**RESPONSES TO REVIEWERS**

**Unstructured Spare Time as an International Predictor of Adolescent Crime**

**(Manuscript ID PONE-D-26-07062)**

______________________________________________________________________________

Dear Editor,

Thank you for your decision and for the opportunity to revise our manuscript. The reviewers’ feedback was insightful and helpful, and we have followed their recommendations in every instance.

As requested by the Managing Editor, we have also reworked the structure of the article, including moving the ethics statement to the Methods section, and formatted the manuscript in accordance with the journal’s guidelines.

We note that, in order to accommodate the revisions suggested by the reviewers, we have had to slightly shorten some sections of the article. We believe the revised manuscript is stronger as a result.

In the remainder of this memo, we outline the revisions made to the manuscript, listing each change alongside the relevant reviewer comment.

REVIEWER 1’S COMMENTS

Reviewer 1 commented that: *“This is an impressive and comprehensive study which draws on survey data from just under 60K young people across 21 countries. It explores the relationship between adolescent’s (aged between 13-17 years old) unstructured spare time and self-reported offending, for both the prevalence (probability of offending) and incidence (scale of that offending) of self-reported offending. The research shows that increased unstructured spare time out of the home is associated with higher levels of offending and is consistent across most countries in the study.”*

**RESPONSE.** We thank the Reviewer for their kind words about our study and appreciate their detailed and thoughtful comments. We respond below to each query raised and have done our best to revise the manuscript in line with the reviewer’s comments and suggestions.

Reviewer 1 observed that: *“The paper addresses the relationship between unstructured spare time and adolescent offending with compelling results. The more unstructured spare time out of the home was associated with greater levels of offending, which makes sense. Especially, where there is also a lack of parental control and coupled with delinquent peers and greater exposure to crime. The model accounts for unstructured spare time at home and unstructured spare time out of the home, both of which increase the odds and scale of offending. The model also accounts for structured spare time at home, which has the opposite effect and reduces the odds and scale of offending. I therefore wondered, for completeness, what about the structured spare time out of the home? On lines 279-280 you say that the survey did not include measures of structured out of home spare time – that is such a shame. But, is there any way this could be approximated given the information we do know? If not, I think there is scope for some discussion to address this more explicitly. The focus of the paper is of course, the unstructured spare time and its effect on adolescent crime but given time is definitive.”*

**RESPONSE.** This is an important consideration and we thank the reviewer for raising it. We agree that including structured out-of-home spare time activities in the analysis would substantially enhance the study’s implications for both theory development and, especially, crime prevention practise during adolescence. Activities such as sports programs or out-of-home after-school activities are not currently included in the analysis, even though they may strongly influence adolescents’ time use and opportunities to engage in crime. We thoroughly reviewed the survey items and found no measures that could reasonably approximate this information. We were unable to revise the analysis in this respect. We nevertheless agree that this is a priority for future research in the area and have therefore added the following explanation to the Discussion section:

“Relatedly, the available data do not capture structured out-of-home activities such as sports participation, clubs, or organized after-school programs. Prior research has shown that engagement in structured out-of-home leisure activities may be linked to lower levels of delinquency [30, 34, 48-51]. Moreover, prior research has also reported that adolescents who are more involved in structured activities—particularly sports—may exhibit weaker associations between unstructured activities and delinquency [49]. Future research should examine how structured out-of-home activities compare with the forms of spare time analyzed here in their association with adolescent offending.” (p. 17)

Reviewer 1 noted that: *“The paper reports the results straight after the introduction, while the materials and methods section features at the end. The paper would benefit from moving the materials and methods section to before the results as with the normal convention of a research paper. This would make the study clearer from the outset and address the questions that arose as I read the results and discussion sections. The materials and methods section did answer all my questions that had but it would aid the interpretation of the results if it had featured earlier.”*

**RESPONSE.** We appreciate the reviewer’s comment. Following this recommendation, the Methods section has now been moved immediately below the Introduction. All subsequent section and reference numbers have been modified accordingly.

Reviewer 1 asked: *“The study uses the International Self-Report Delinquency Study, a school-based survey instrument to measure self-reported offending. The supplementary material notes that the sample is not nationally representative and comparisons should be interpreted between cities rather than entire countries. For clarity, this could be made explicit within the main paper.”*

**RESPONSE.** This is an important consideration that was not sufficiently clear in the main manuscript. We have now added the following sentence to the Methods section to clarify this point:

“Given that samples were drawn from urban areas only, cross-national comparisons reflect differences between sampled cities rather than entire countries.” (p. 6)

Reviewer 1 commented that: *“The study uses a Quasi-Poisson model to model the offending counts which is sensible. Out of curiosity, did the authors consider using a Negative-Binomial model? It would be interesting to assess the model fit and compare estimates from these two models.”*

**RESPONSE.** We thank Reviewer 1 for raising this point. We had considered using a Negative Binomial regression to estimate crime counts, but selected a quasi-Poisson specification because it aligned better with the overdispersed nature of the data. Following the reviewer’s recommendation, however, we replicated the main analysis using a Negative Binomial model, and the results were substantively unchanged. We have added these robustness checks as Appendix S7 and included the following sentences in the main manuscript:

“We additionally estimated negative binomial regression models to assess the sensitivity of the results to alternative count model specifications and present these as robustness checks in Appendix S7.” (p. 8)

“The Negative Binomial models, whose results can be consulted in Appendix S7, produced substantively equivalent results to the main analyses.” (pp. 11-12)

REVIEWER 2’S COMMENTS

Reviewer 2 commented that: *“The manuscript “Unstructured Spare Time as an International Predictor of Adolescent Crime” examines the relationship between adolescents’ time use and self-reported offending across 21 countries using a very large and valuable dataset. The paper addresses an important question in criminology and makes a potentially significant empirical contribution by moving beyond the usual single-country focus that dominates the literature on leisure and delinquency.*

*Overall, I find the paper interesting, well-executed in many respects, and clearly positioned within existing research, but I also believe that several conceptual and methodological issues need to be addressed before it can be considered for publication. In its current form, the manuscript tends to overinterpret its findings and would benefit from greater precision regarding what can (and cannot) be concluded from the data.*

*One of the strongest aspects of the paper is the dataset itself. The use of the ISRD4 data, with more than 58,000 adolescents across a diverse set of countries, provides a level of external validity that is rarely achieved in this line of research. The consistency of the results across countries is also compelling and, in principle, supports the authors’ argument that unstructured spare time—particularly out-of-home—is a robust correlate of offending.”*

**RESPONSE.** We thank Reviewer 2 for their positive assessment of our study and for their detailed and thoughtful feedback. Below, we respond to each point raised and have revised the manuscript accordingly to reflect the reviewer’s comments and suggestions.

Reviewer 2 observed: *“At the same time, the manuscript would benefit from a more cautious and theoretically grounded interpretation of these findings. The current framing sometimes suggests a level of causal inference that is not supported by the cross-sectional design, and some measurement and modeling choices require further clarification and justification.*

*The most important issue concerns the way the results are interpreted. Although the authors briefly acknowledge the cross-sectional nature of the data, several parts of the manuscript—particularly the Abstract, Discussion, and the simulation section—go beyond associational claims and imply causal effects. For example, the simulations suggesting that reducing unstructured spare time would lead to measurable reductions in offending rely on assumptions that cannot be justified with the current design.*

*This is problematic because alternative explanations are clearly plausible. Adolescents who are already involved in delinquency may be more likely to spend time in unstructured settings (selection effects), and unobserved factors such as family environment, neighborhood context, or personality traits could drive both time use and offending. While the inclusion of several control variables is a strength, it does not resolve these fundamental identification issues.*

*I would strongly encourage the authors to reframe the paper consistently in associational terms, reduce causal language throughout, and substantially qualify the interpretation of the simulation results. As it stands, the policy implications are too strong relative to the evidentiary basis.”*

**RESPONSE.** We thank Reviewer 2 for raising this important point and agree with the concern expressed. We acknowledge that several parts of the original manuscript employed language that could be interpreted as implying a stronger level of causal inference than is warranted by the cross-sectional design. We have thoroughly revised the manuscript throughout to ensure that findings are framed consistently in associational rather than causal terms. We have replaced terms such as “effects,” “predictors,” and “risk factors” where appropriate, and clarify that the simulation analyses represent model-based projections rather than causal estimates of intervention impacts. We have also qualified the policy implications to ensure that they are presented more cautiously and proportionately to the design of the study. We provide below several examples of the revised sentences:

“This study investigates whether unstructured spare time is a significant correlate of self-reported offending among adolescents in multiple countries.” (p. 3)

“The estimated association of self-control is similar in size to that of unstructured out-of-home spare time; however, it operates in the opposite direction, being negatively associated with individual crime involvement.” (p. 11)

“All countries except Lithuania demonstrate significant and strong positive associations between unstructured out-of-home spare time and either offending prevalence or incidence.” (p. 13)

“For context, having delinquent peers is the only other variable in our national-level analyses that shows statistically significant associations with self-reported offending in all but one country.” (p. 14)

“Interventions that seek to redirect unstructured spare time, particularly in public, outdoor settings, and expand access to structured, supervised, and meaningful activities may represent promising avenues for prevention, although their effectiveness should be evaluated using stronger causal designs.” (p. 18)

In addition to these revisions, we have sharpened the conceptual framing in the Discussion's theoretical passage to make explicit that the paper's contribution is interpretive and theoretical rather than causal: the cross-national consistency of the association is presented as establishing a cross-cultural empirical regularity that places new demands on existing theoretical frameworks, rather than as causal evidence for intervention. This reinforces the associational stance emphasized throughout this response.

Reviewer 2 asked: *“A second major concern relates to how “unstructured spare time” is operationalized. The index combines a wide range of activities, some of which are conceptually quite different and may even overlap with deviant behavior itself. For example, including “skipping class” within the measure raises questions, as this is arguably already a form of rule-breaking rather than a neutral leisure activity. Similarly, the inclusion of heterogeneous online activities (from social media use to darknet browsing) blurs important distinctions.*

*This raises the possibility of conceptual and empirical overlap between the independent variable and the outcome, which could inflate the observed associations. At a minimum, the authors should provide a clearer justification for the inclusion of each component and discuss the implications of this measurement strategy.”*

**RESPONSE.** This is an important consideration and we appreciate the reviewer raising this point. In our view, here the reviewer’s comment raises two separate concerns: first, that our measures of unstructured spare time combine a range of different activities that may blur important distinctions both in their nature and in their relationship with crime; and second, that our measures of unstructured spare time include activities that may themselves be considered deviant and therefore may conceptually overlap with the dependent variables under study. We address these two concerns separately below.

Regarding the first point (i.e., that our measures of unstructured spare time combine a range of different activities that may mask important heterogeneity), we agree that this is an important consideration. It is indeed possible that our estimates average over activities with different implications for crime and crime prevention practice. However, the overall aim of the study was to assess whether unstructured spare time activities are, on average, more strongly related to self-reported offending across countries than structured activities. Analysing each activity separately would require a substantially expanded set of analyses and a different analytical focus from that of the present study. While we consider this beyond the scope of the present study, we acknowledge it as an important priority for future research and have added the following sentences to the Discussion section to clarify this point:

“Our measures of time use combine numerous indicators into overarching groups of structured and unstructured behaviors, which may potentially obscure internal heterogeneity across specific activities [48, 49]. Browsing social media, for example, may have less detrimental effects than navigating the darknet, and the level of formal control may vary between activities such as hanging about in the streets and attending parties. While assessing the relationship between each specific activity and offending is beyond the scope of this article, we highlight this as an important area for future research with potentially significant implications for crime prevention practice.” (p. 17)

Regarding the second point (i.e., that some items included in our unstructured spare time measures may themselves be considered deviant), we also agree that this is an important consideration and have re-run the analyses after excluding those measures of unstructured time use that may be considered deviant and thus conceptually close to the crime outcome (i.e., truancy and navigating the darknet). We present these results as a robustness check in the new Appendix S9. Results are essentially unaffected after excluding these items from the unstructured spare time measures. We have noted this in the manuscript with the sentence below and provide further details in Appendix S9.

“To ensure that our results were not affected by the inclusion of spare time activities that may themselves be considered deviant rather than merely unstructured (i.e., truancy and navigating the darknet), we re-estimated the main analyses after excluding these items from the unstructured spare time measures. The results, shown in Appendix S9, remain substantively equivalent.” (p. 17)

Reviewer 2 commented: *“The cross-national scope is a major strength, but it also introduces important challenges that are not fully addressed. The analysis assumes that key constructs—such as unstructured time and self-reported offending—are comparable across countries. However, differences in cultural norms, reporting practices, and survey interpretation may affect both variables.*

*While the inclusion of country fixed effects helps control for average differences between countries, it does not address measurement equivalence. Without some discussion (or testing) of measurement invariance, it is difficult to assess whether the observed cross-national consistency reflects a substantive pattern or, at least in part, similarities in measurement artifacts.*

*I would therefore recommend that the authors discuss this issue of cross-national comparability, even if formal invariance testing is not feasible within the scope of the paper.”*

**RESPONSE.** We thank Reviewer 2 for raising another important point. We agree that cross-national comparability is a key consideration in international survey research and that differences in cultural norms, language, and survey interpretation may influence responses across countries. Although formal measurement invariance testing is less applicable to the key variables under study in the present study because our measures of unstructured spare time and offending are constructed as summative behavioral indices rather than latent reflective scales, we acknowledge that this does not eliminate broader concerns regarding comparability across national contexts. We have therefore added text to the manuscript discussing this limitation and noting that some degree of cross-national variation may reflect differences in interpretation or measurement rather than substantive behavioral differences alone.

“Importantly, although our analyses draw on cross-nationally harmonized survey instruments, cross-national comparisons should be interpreted with appropriate caution. Differences in cultural norms, translation, and survey interpretation may affect the comparability of responses across national contexts. As such, some observed cross-national variation may reflect measurement differences in addition to substantive behavioral differences.” (pp. 16-17)

Reviewer 2 asked: *“Relatedly, the modeling approach could be better justified. The data have a clear hierarchical structure (students nested within schools and countries), yet the analysis relies on clustered standard errors rather than multilevel models. While this is not necessarily incorrect, the choice should be explicitly justified, especially given the comparative focus of the study.”*

**RESPONSE.** We thank Reviewer 2 for another important comment. We agree that the hierarchical structure of the data warrants explicit justification of the modeling strategy. Our analytical approach combines pooled models with country fixed effects and school-clustered standard errors, alongside separate country-specific models, as a way to account for the hierarchical structure of the data while aligning with the overall aims of the study. We follow this approach because the objective of the study is both to estimate pooled associations and to examine heterogeneity across national samples. This strategy was preferred over multilevel random-effects models because it allows us to compare country-specific estimates directly rather than estimate them under a partial-pooling framework that may shrink national estimates toward an overall mean. Clustering standard errors at the school level accounts for within-school dependence among respondents, while country fixed effects adjust for unobserved between-country differences in the pooled models. We have now further clarified this rationale in the Methods section:

“To account for the nested structure of the data (students within schools), standard errors are clustered at the school level, and all p-values are based on robust sandwich estimators [38]. Country fixed effects are included to adjust for unobserved between-country differences [39]. We estimate the models for all respondents first, and second for each country independently. This staged approach enables us to examine national associations directly and compare them across countries without constraining country-level estimates to follow a common distribution.” (p. 8)

Reviewer 2 asked: *“The use of pairwise t-tests with Benjamini–Hochberg correction is appropriate, but the implementation is not fully described (e.g., number of tests, whether correction is applied globally).”*

**RESPONSE.** We thank the reviewer for this helpful observation. We have now clarified both the number of tests conducted and that the Benjamini-Hochberg correction was applied independently within each national sample:

“In total, 63 pairwise comparisons were conducted (three within each national sample), with the Benjamini-Hochberg correction applied separately within each country.” (p. 10)

Reviewer 2 asked: *“The manuscript does not specify whether assumptions underlying the t-tests (particularly homogeneity of variances) were assessed, or whether Welch corrections were used.”*

**RESPONSE.** We thank Reviewer 2 for this helpful observation. The original pairwise comparisons were estimated using the default implementation. Following the reviewer’s recommendation, we re-estimated all pairwise comparisons using Welch-type tests that do not assume homogeneity of variances. The results were substantively unchanged.

“Welch-type pairwise t-tests are used to assess whether the differences displayed in Fig 1 are statistically significant, with p-values adjusted for multiple testing using the Benjamini-Hochberg procedure.” (p. 10)

*Reviewer 2 asked:* *“The variable capturing “openness in honestly reporting” is a potentially valuable addition, but its construction, validity, and relationship to other variables (such as morality or self-control) are not sufficiently explained.”*

**RESPONSE.** We agree that the content of this variable was not sufficiently explained in the manuscript. We have now added the following sentence to clarify the question wording:

“Openness to respond sincerely is measured using the item “imagine you had shoplifted; do you think that you would have said so in this survey?” [36, 37].” (p. 7)

Moreover, following the reviewer’s recommendation, we assessed its relationship with other key variables, including offending incidence (Spearman’s rank correlation = 0.14, p < 0.001), morality (rho = 0.08, p < 0.001) and self-control (rho = 0.01, p = 0.08), showing that although these variables are correlated, the associations are not strong enough to raise multicollinearity concerns.

*Reviewer 2 noted:* *“In sum, this is a strong and potentially impactful paper that makes excellent use of a uniquely rich and large-scale cross-national dataset to address a highly relevant question in criminology. The empirical analyses are carefully conducted, and the consistency of the findings across countries is particularly compelling. The manuscript clearly demonstrates the promise of a time-use perspective for understanding adolescent offending and offers insights that are likely to be of broad interest to researchers and policymakers alike.*

*That said, the paper would benefit from some further refinement, particularly in clarifying aspects of measurement and modeling, adopting a slightly more cautious interpretation of the findings, and engaging more explicitly with issues of cross-national comparability. These are, in my view, constructive improvements that would strengthen an already valuable contribution.”*

**RESPONSE.** We once again thank Reviewer 2 for their thoughtful and detailed comments, which have helped us improve the manuscript and its clarity. We hope that the responses provided above satisfactorily address the reviewer’s requests and considerations.
